# Identities of P2 and P3 Residues of H-2K^b^-Bound Peptides Determine Mouse Ly49C Recognition

**DOI:** 10.1371/journal.pone.0131308

**Published:** 2015-07-06

**Authors:** Elsa A. Marquez, Kevin P. Kane

**Affiliations:** Medical Microbiology and Immunology, University of Alberta, Edmonton, Alberta, Canada; University of East London, UNITED KINGDOM

## Abstract

Ly49 receptors can be peptide selective in their recognition of MHC-I-peptide complexes, affording them a level of discrimination beyond detecting the presence or absence of specific MHC-I allele products. Despite this ability, little is understood regarding the properties that enable some peptides, when bound to MHC-I molecules, to support Ly49 recognition, but not others. Using RMA-S target cells expressing MHC-I molecules loaded with individual peptides and effector cells expressing the ectodomain of the inhibitory Ly49C receptor, we found that two adjacent amino acid residues, P2 and P3, both buried in the peptide binding groove of H-2K^b^, determine mouse Ly49C specificity. If both are aliphatic residues, this is supportive. Whereas, small amino acids at P2 and aromatic amino acids at the P3 auxiliary anchor residue are detrimental to Ly49C recognition. These results resemble those with a rat Ly49 where the identity of a peptide anchor residue determines recognition, suggesting that dependence on specific peptide residues buried in the MHC-I peptide-binding groove may be fundamental to Ly49 peptide selectivity and recognition.

## Introduction

Natural killer (NK) cells play an important role in the clearance of tumor cells and virally infected cells [1–3]. NK cell activity depends on the integration of signals mediated by activating and inhibitory receptors. Examples of inhibitory receptors include Ly49s in rodents and Killer Ig-related (KIR) receptors in humans [4]. Inhibitory receptors expressed on NK cells interact with peptide-bound class I major histocompatibility complex (MHC-I) molecules to maintain NK cell tolerance to self. Although structurally distinct, Ly49 and KIR have analogous functions, and can recognize MHC-I in an allele specific manner [5].

Similarly, Ly49 and KIR receptors can be peptide selective in their recognition of MHC-I-peptide complexes [6–10]. In other words, some MHC-I bound peptides, but not all, support receptor recognition. This suggests that Ly49 and KIR can have a level of ligand specificity that extends beyond simply detecting the presence or absence of an MHC-I allele product, but includes the MHC-I bound peptide as well. The peptide repertoire for MHC-I binding can be modified by viral infection and tumorigenic transformation [11,12]. NK cells may subsequently detect such modifications as changes in the self peptide pool normally displayed on MHC-I, thus altering the balance of inhibitory and activating signals, and consequently altering NK cell activity.

Given that important processes in the life of an NK cell can depend on Ly49 and MHC-I association, such as NK cell education and NK cell effector functions, it is of interest to examine the identities of peptide residues that give rise to successful ligand and receptor interaction. Inhibitory Ly49 receptors that are MHC-I peptide selective include mouse Ly49C and Ly49I, as well as rat Ly49i2. Discrimination of H-2K^d^ by Ly49I was first shown by Hanke *et al*, where only certain peptides bound to H-2K^d^ supported receptor interaction with ligand [7]. Similarly, Ly49C has been demonstrated to selectively associate with H-2K^b^ depending on the peptide bound [13]. Moreover, Kärre and co-workers provided evidence that the amino acid at position 7 (P7) of the H-2K^b^ bound peptide partially influenced Ly49C and H-2K^b^ association [13]. Studies in our laboratory showed that recognition between a rat Ly49, Ly49i2 and cognate ligand RT1-A1^c^ is also peptide dependent and specific to the peptide anchor residue at P2 of the RT1-A1^c^ peptide bound [8]. As demonstrated by crystallography studies, Ly49 recognition of MHC-I molecules occurs at a site underneath the peptide binding groove, a region that can be affected by peptide anchor residues, possibly affecting the topology of the MHC-I site for Ly49 association [14]. Utilizing Ly49C and H-2K^b^ association as a model, we investigated the influence of anchor residues in determining their interaction.

Peptide dependent recognition of H-2K^b^ by Ly49C was addressed by examining lysis of the TAP-2 deficient RMA/S target cells expressing H-2K^b^ bound with different peptides, by effector RNK-16 cells stably transfected with the Ly49C ectodomain linked to the transmembrane and intracellular domain of the Ly49W activating receptor. Herein, we show that the identity of the peptide auxiliary anchor residue P3 correlates with Ly49C recognition. Moreover, in addition to P3, we identified the adjacent P2 residue, that also docks in the peptide binding groove, to significantly influence Ly49C peptide selectivity in recognition of H-2K^b^. In particular, we found that Ly49C and H-2K^b^ interaction is supported by non-polar aliphatic residues at P3 and bulky non-polar aliphatic residues at P2. This indicates that not only in rat, but also in mice, Ly49 recognition can be determined by peptide residues bound in specific buried locations in the peptide binding groove, and suggests this may be a fundamental feature of Ly49 recognition of MHC-I ligands.

## Materials and Methods

### Cell lines and peptides

The TAP-2 deficient RMA-S mouse T lymphoma cell line was obtained from Dr. W. Jefferies (University of British Columbia, Vancouver, Canada) [15,16]. RNK-16 is a spontaneous F344 rat strain NK cell leukemia cell line [17]. Both cell lines were maintained in RPMI supplemented with 10% FCS, L-glutamine, penicillin, streptomycin, and 50 μM 2-mercaptoethanol (2-ME), unless otherwise indicated. Synthetic peptides were purchased from GenScript, each with 98% purity or higher and dissolved in DMSO.

### Monoclonal antibodies and cell staining

The H-2K^b^-specific mAb clone AF6-88.5.5.3 coupled to Allophycocyanin (APC) was purchased from eBioscience. Incubation with AF6-88.5.5.3-APC specific antibodies was performed at a final concentration of 2μg/ml for 15 minutes at 4°C in Phosphate Buffered Saline (PBS) containing 2%FCS and 5mM EDTA (Staining Buffer). Following antibody incubation, cells were washed twice with Staining Buffer before fixing cells in PBS buffer containing 5mM EDTA and 1% formaldehyde. Stained and fixed samples were analyzed in a FACSCanto cell analyzer (BD Biosciences).

The RNK-16 transfectants were incubated with rat IgG (Sigma) for 30 minutes at 4°C to block nonspecific antibody binding to Fc receptors before staining. The 4L03311 hybridoma, producing the Ly49C specific mAb, was a gift from Suzanne Lemieux (Université du Québec, Laval, Québec, Canada). The Ly49C specific 4L03311 mAb was prepared by ammonium sulfate precipitation and PBS dialysis of the supernatant from the hybridoma grown in protein free media. Fluorescein isothiocyanate (FITC) coupled secondary rat-anti-mouse antibody was purchased from Cedarlane. Incubation with primary Ly49C specific mAb was done at a final concentration of 50μg/ml for 15 minutes at 4°C in Staining Buffer. Following 4L03311 mAb incubation, cells are washed twice before secondary antibody is added at a 1:50 dilution in Staining Buffer. After incubation with secondary antibody, cells are washed twice with Staining Buffer before fixing cells in PBS buffer containing 5mM EDTA and 1% formaldehyde. Stained and fixed samples were analyzed in a FACSCanto cell analyzer (BD Biosciences).

### RMA/S stabilization assay

RMA/S cells were incubated at 26°C in AIMV serum free media (Invitrogen) supplemented with 50μM mercaptoethanol and 1mM glutamine for 16 hours [18]. Incubation with individual peptides was conducted for 15 minutes in the presence of Brefeldin A (BFA, Invitrogen) to block transport of newly synthesized MHC-I molecules to the cell surface [19,20]. RMA/S cells were washed three times using AIMV media to discard any excess peptide and were further incubated at 37°C for 4 hours to test stabilization of H-2K^b^. Peptide binding to H-2K^b^ was analyzed by surface staining of H-2K^b^ in a FACSCanto cell analyzer (BD Biosciences).

### Generation of RNK-16 cells expressing chimeric Ly49W/C

The cDNA encoding the intracellular and transmembrane domain of Ly49W^NOD^ (residues 1–66) fused with the extracellular domain of Ly49C^C57BL/6^ (residues 67–261) was generated using two rounds of PCR amplification. For the first round, we amplified the transmembrane and cytoplasmic region of Ly49W^NOD^ using the 5’ primer 5’-CCGCTCGAGATGAGTGAGCAGGAGGTCACTTTC that included a XhoI restriction site; and the 3’ primer 5’-AGTTTTTTTTGTTGATCATACTGAAAAATTTTTGTC ACAAGCACTGAGACAAT that included a overhang sequence to bind the 5’ extracellular region of Ly49C^C57BL/6^. The PCR product was run on an agarose gel, purified and used as the 5’ primer for the second round of PCR amplification with the 3’ primer 5‘TGTGTTGATTATACTGAAAAATCTTTGTCACAAGCACTGAGACAAT that included the EcoRI restriction site. The final PCR product, chimeric Ly49W/C, was subcloned into the pCIneo vector and then into BSRαEN for transfection of RNK-16 cells. Stable RNK-16 cell transfectants expressing the chimeric Ly49W/C receptor (RNK.49W/C) were produced as described [8] and maintained in RPMI media supplemented with 10%FCS, L-glutamine, penicillin, streptomycin and 2-ME under antibiotic selection using 10mg/ml G418. (Geneticin, invitrogen). Expression of chimeric Ly49W/C on RNK-16 cells was confirmed by cell surface staining with the 4L03311 (anti-Ly49C) mAb and analyzed by flow cytometry using FACSCanto cell analyzer (BD Biosciences) gating on live cells.

### Cytotoxicity Assays

RMA-S cells were cultured for 16 hours at 26°C in serum free AIMV media, supplemented with L-glutamine and 2-ME. The cells were then incubated with peptide for 15 minutes and 10μg/ml of BFA at 26°C, followed by three washes to eliminate unbound peptide. Peptide-loaded RMA-S cells were labeled with 100–150 μCi of ^51^Cr (^51^Chromium Radionuclide, Perkin Elmer) for 1 hour at 37°C and washed extensively. Effector RNK.49W/C cells were incubated without selective antibiotic G418 (Geneticin, Invitrogen) 48 hours prior to the assay and washed using serum free AIMV media. Cytotoxicity assays were carried out for 4 hours at 37°C with varying effector to target (E:T) cell ratios in 96-well V-bottom microtitre plates. Following the 4 hour incubation, microtitre plates were centrifuged for 4 minutes and 25μl of supernatant were transfered to a 96-well flexi-plate with 100μl of scintillant (OptiPhase ‘SuperMix’, Wallac, Loughborough, England). Flexi-plates were incubated in a plate shaker for 10 minutes at 900RPM, and further analyzed in a beta counter (MicroBeta, Wallac, Loughborough, England). Percent specific lysis was calculated as [(experimental release)–(spontaneous release)/(total release)–(spontaneous release)] x 100. All cytotoxicity assays were performed in triplicate in a minimum of three independent experiments.

### Statistical Analysis

The Mean, SD and *p* values were calculated using GraphPad Prism Version 5 (GraphPad Software). Peptide dependent statistically significant decrease in Ly49W/C recognition of H-2K^b^ was determined by comparing the percent specific lysis between the positive control for recognition, RMA/S cells loaded with the SIINFEKL peptide, and RMA/S cells loaded with peptides tested for recognition, or no peptide. The *p* values were determined from percent specific lysis values at the 12.5:1 E:T ratio using a two-tailed paired *t*-test with 95% confidence interval (*p < 0.05, **p < 0.005).

### Structural Analysis

Molecular graphics were compiled using the UCSF Chimera software (Resource for Biocomputing, Visualization, and Informatics, University of California, San Francisco (http://www.cgl.ucsf.edu/chimera). Protein Data Bank (PDB) ID codes used were 1VAC for H-2K^b^-SIINFEKL, 2VAA for H-2K^b^-RGYVYQGL and 3C8K for H-2K^b^-SIINFEKL-Ly49C co-crystal structure.

### Prediction of peptide binding affinities to H-2K^b^ using NetMHCpan

The server NetMHCpan was used to predict the affinity between specific MHC-I molecules and peptides bound that are expressed in nM IC_50_ values [21]. Options to set a threshold for strong and weak binders were set to IC_50_ of 10nM and IC_50_ of 500nM, respectively.

## Results and Discussion

### Identity of the peptide auxiliary anchor residue at P3 correlates with Ly49C recognition of H-2K^b^

Previous studies have shown that association between Ly49C and H-2K^b^ is supported by the OVA peptide SIINFEKL, but this interaction is not supported by the vesicular stomatitis virus (VSV) peptide RGYVYQGL and the synthetic peptide AAYAYAAL [13,22]. We tested additional peptides in order to have a larger pool of H-2K^b^-peptides to compare identities of anchor residues and Ly49C recognition (Tables 1 and 2). The peptides used in this experiment are known to bind H-2K^b^ from peptide elution studies, with some of these peptides also tested for T cell receptor recognition [23–32].

**Table 1:** Sequence and origin of H-2K^b^ specific peptides bearing non-polar aliphatic auxiliary anchor residues.

Peptide Sequence	Peptide origin
SI**I**N**F**EK**L**	Chicken Ovalbumin (256–264)
KI**I**T**Y**RN**L**	PCI domain-containing protein 2 (318–325)
KV**I**T**F**ID**L**	GTP binding protein I (246–253)
YA**M**I**Y**RN**L**	E3 ubiquitin-protein ligase mdm2 (100–107)

Bold indicates primary anchor residue, bold and underlined indicates P3 auxiliary anchor residue.

**Table 2:** Sequence and origin of H-2K^b^ specific peptides bearing aromatic auxiliary anchor residues.

Peptide Sequence	Peptide origin
AN**Y**D**F**IC**V**	MMTV env gp70 (446–453)
RG**Y**V**Y**QG**L**	Vesicular Stomatitis Virus NP (52–59)
IS**F**K**F**DH**L**	F-actin capping protein alpha1 (93–100)
IN**F**D**F**PK**L**	RNA helicase p54 (407–414)
AA**Y**A**Y**AA**L**	Synthetic peptide

Bold indicates primary anchor residue, bold and underlined indicates P3 auxiliary anchor residue.

**Fig 1:**
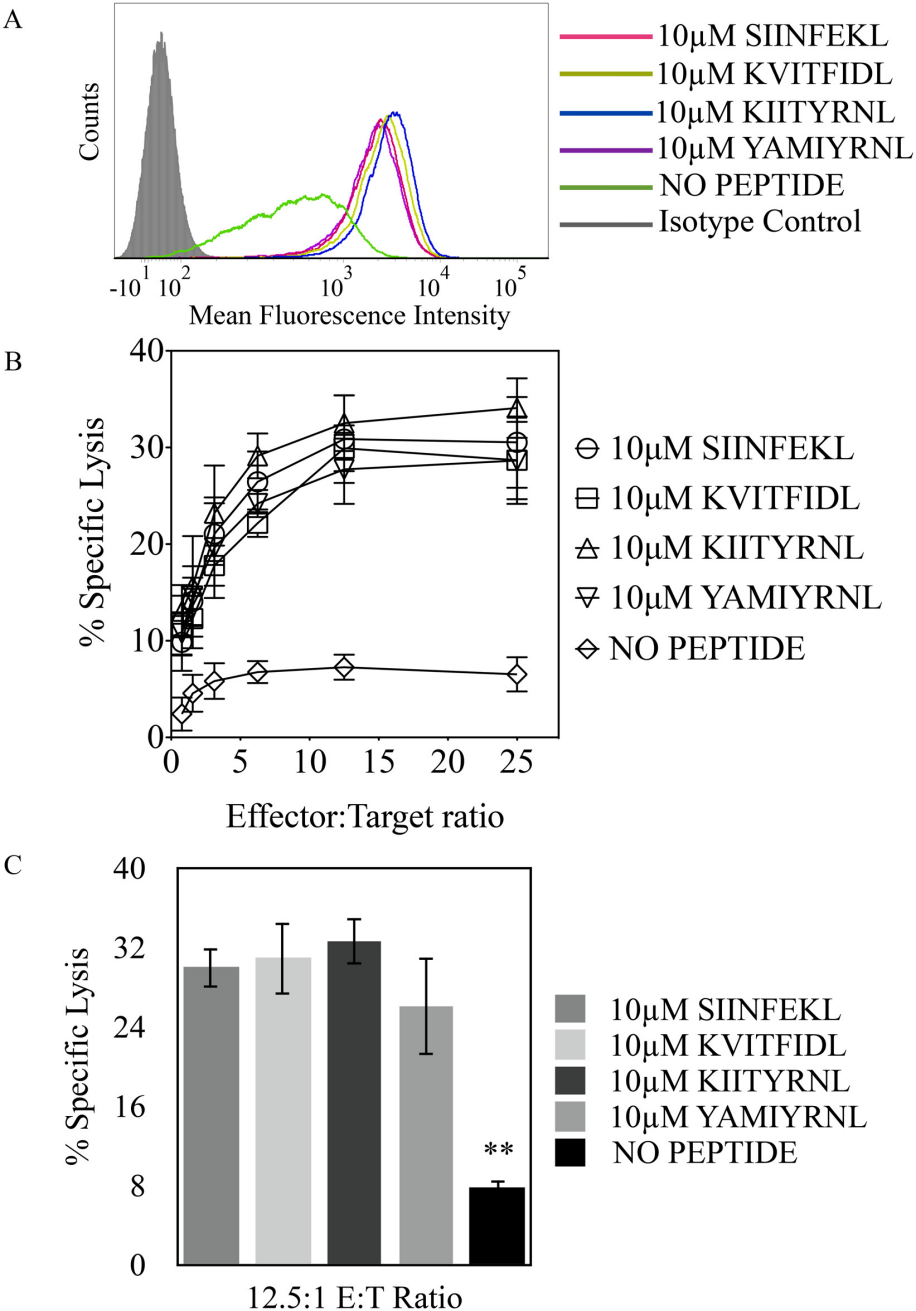
Ly49C peptide dependent recognition of H-2K^b^ is supported using peptides with non-polar aliphatic auxiliary anchor residues. **(A)** RMA-S cell surface expression of H-2K^b^ when incubated with the indicated peptides, or no peptide. Shaded histogram corresponds to isotype control. **(B)** Percent specific lysis of RMA-S cells incubated with peptides bearing aliphatic R groups at P3, in the presence of RNK.49W/C effector cells. **(C)** Statistically significant changes in Ly49W/C recognition of H-2K^b^, with respect to the positive control for RNK.49W/C recognition (SIINFEKL-loaded RMA/S cells), were conducted at the 12.5:1 E:T ratio from cytotoxicity assays (**p < 0.005). Results plotted are the mean of three independent experiments with error bars representing SD.

**Fig 2:**
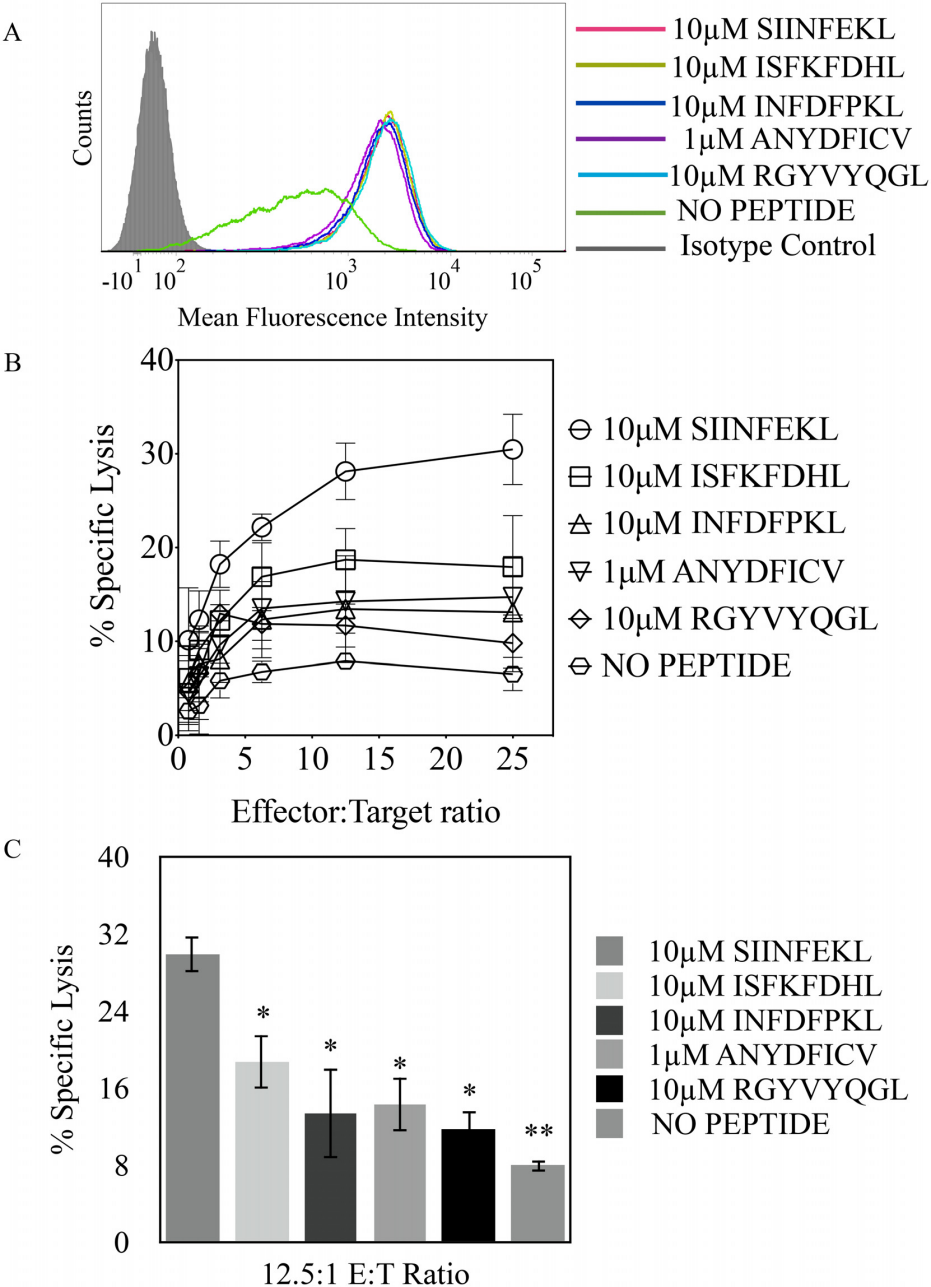
Ly49C peptide dependent recognition of H-2K^b^ decreases with peptides bearing non-polar aliphatic auxiliary anchor residues. **(A)** RMA-S cell surface expression of H-2K^b^ when incubated with the indicated peptides, or no peptide. Shaded histogram corresponds to isotype control. **(B)** Cytotoxicity assay using RMA-S cells loaded with peptides that have aromatic R groups at P3 co-incubated with RNK.49WC effector cells. **(C)** Statistically significant changes in Ly49W/C recognition of H-2K^b^, with respect to the positive control for RNK.49W/C recognition (SIINFEKL-loaded RMA/S cells), were performed at the 12.5:1 E:T ratio from cytotoxicity assays (*p < 0.05, **p < 0.005). Results plotted are the mean of three independent experiments with error bars representing SD.

From the initial set of peptides used in this study we found that along with SIINFEKL, peptides KIITYRNL, KVITFIDL, and YAMIYRNL stabilized H-2K^b^ on RMA/S cells (Fig 1A) and similarly supported Ly49W/C recognition (Fig 1B and 1C). On the other hand, although capable of yielding similar H-2K^b^ expression on RMA/S cells (Fig 2A), peptides ANYDFICV, INFDFPKL, and ISFKFDHL did not, or poorly supported H-2K^b^ and Ly49W/C interaction, (Fig 2B and 2C) resembling RGYVYQGL and AAYAYAAL peptides previously shown to be unsupportive of H-2K^b^ and Ly49C interaction [22]. Peptides that supported H-2K^b^ and Ly49W/C interaction, SIINFEKL, KIITYRNL, KVITFIDL and YAMIYRNL have the same P8 residues, conserved aromatic P5 residues and non-polar aliphatic P3 residues. While peptides that did not confer Ly49W/C and H-2K^b^ interaction, ANYDFICV, INFDFPKL, ISFKFDHL, RGYVYQGL and AAYAYAAL have similar P8 and P5 residues, but have aromatic residues at P3. Therefore, given the correlation between the identity of auxiliary anchor residue at P3 and Ly49W/C recognition, as noted in Table 3, we hypothesized that peptides bearing non-polar aliphatic residues at P3 are supportive of receptor and ligand interaction, while aromatic residues at P3 are detrimental for H-2K^b^ and Ly49W/C association.

**Table 3:** Peptide dependent Ly49W/C recognition of H-2K^b^.

Peptide Origin	H-2K^b^ bound peptide sequence								% specific lysis (Ly49C recognition)
	1	2	3	4	5	6	7	8	
PCI domain protein	K	I	**I**	T	**Y**	R	N	**L**	HIGH
Chicken Ovalbumin (256–264)	S	I	**I**	N	**F**	E	K	**L**	HIGH
E3 ubiquitin-protein ligase	Y	A	**M**	I	**Y**	R	N	**L**	HIGH
GTP binding protein 1	K	V	**I**	T	**F**	I	D	**L**	HIGH
F-actin capping protein	I	S	**F**	K	**F**	D	H	**L**	MED
MMTV env gp70	A	N	**Y**	D	**F**	I	C	**V**	MED
RNA helicase p54 (407–414)	I	N	**F**	D	**F**	P	K	**L**	LOW
VSV NP peptide	R	G	**Y**	V	**Y**	Q	G	**L**	LOW
Synthetic K^b^ binding peptide	A	A	**Y**	A	**Y**	A	A	**L**	LOW

Peptides containing non-polar aliphatic auxiliary anchor residues at P3 support Ly49W/C association with H-2K^b^ as demonstrated by cytotoxicity assays. On the other hand peptides with aromatic residues at P3 are not highly supportive and mostly poorly supportive of Ly49W/C and H-2K^b^ interaction. For peptide amino acid sequence, bold indicates primary anchor residues at P5 and P8, bold and underlined indicates P3 auxiliary anchor residue.

### Aliphatic residues at P3 are supportive of Ly49W/C recognition of H-2K^b^

**Fig 3:**
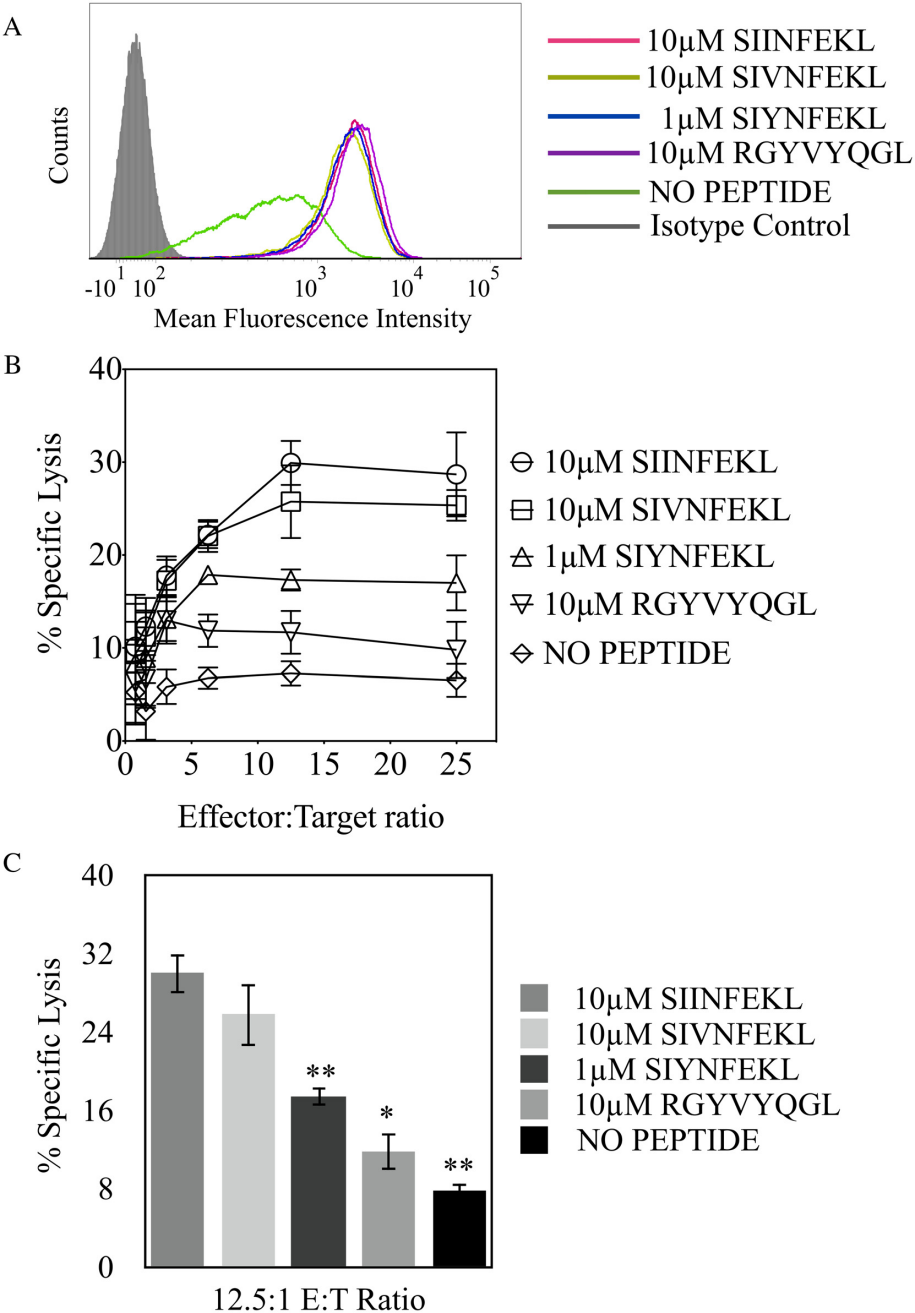
Peptide amino acid substitution demonstrates that auxiliary anchor residue P3 is an important factor in determining Ly49C recognition. **(A)** Expression of H-2K^b^ on RMA/S cells co-incubated with the listed peptides **(B)** Cytotoxic assay using RNK.49W/C effector cells and RMA-S targets incubated with SIINFEKL or the indicated SIINFEKL variants (SIVNFEKL and SIYNFEKL) and RGYVYQGL **(C)** Statistically significant changes in Ly49W/C recognition of H-2K^b^, with respect to the positive control for RNK.49W/C recognition (SIINFEKL-loaded RMA/S cells), were conducted at the 12.5:1 E:T ratio from cytotoxicity assays (*p < 0.05, **p < 0.005). Results plotted are the mean of three independent experiments with error bars indicating SD.

In order to corroborate auxiliary anchor residue P3 directed recognition, we considered an Ala substituted SIINFEKL variant at P3 to directly examine Ly49W/C recognition. However, the SIINFEKL analog bearing Ala at P3 does not stably bind H-2K^b^ compared to the unchanged SIINFEKL peptide, as previously reported [33]. Therefore, we tested recognition by substituting the identity of residues at P3 between RGYVYQGL peptide, that does not confer Ly49C and H-2K^b^ interaction, and SIINFEKL, a peptide supportive of Ly49C interaction with H-2K^b^, creating the SIYNFEKL peptide. In addition, we tested a SIINFEKL variant with Val at P3, similar to Ile, that can allow us to examine preferred residue chemistry at P3 for Ly49W/C and H-2K^b^ association. In parallel to killing assays, we also carried out RMA/S stabilization assays with peptides SIVNFEKL and SIYNFEKL, as well as with control peptides, where all peptides yielded H-2K^b^ expression at similar levels (Fig 3A). Substituting Val for Ile at P3, SIVNFEKL, made no significant difference to H-2K^b^ recognition, as observed in cytotoxicity experiments; however, exchange of a Tyr for Ile, SIYNFEKL, partially reduced H-2K^b^ recognition as compared to the RGYVYQGL peptide or no peptide (Fig 3B and 3C). These results indicated that aliphatic residues Val or Ile at P3 are fully supportive of, while the aromatic and polar Tyr is detrimental to, Ly49W/C interaction with H-2K^b^. The substitution of Tyr for Ile at P3 in SIINFEKL did not completely disrupt recognition, indicating that additional residue(s) may contribute to Ly49C and H-2K^b^ interaction. Nevertheless, the identity of the P3 auxiliary anchor residue of bound peptide is an important factor in H-2K^b^ recognition.

**Fig 4:**
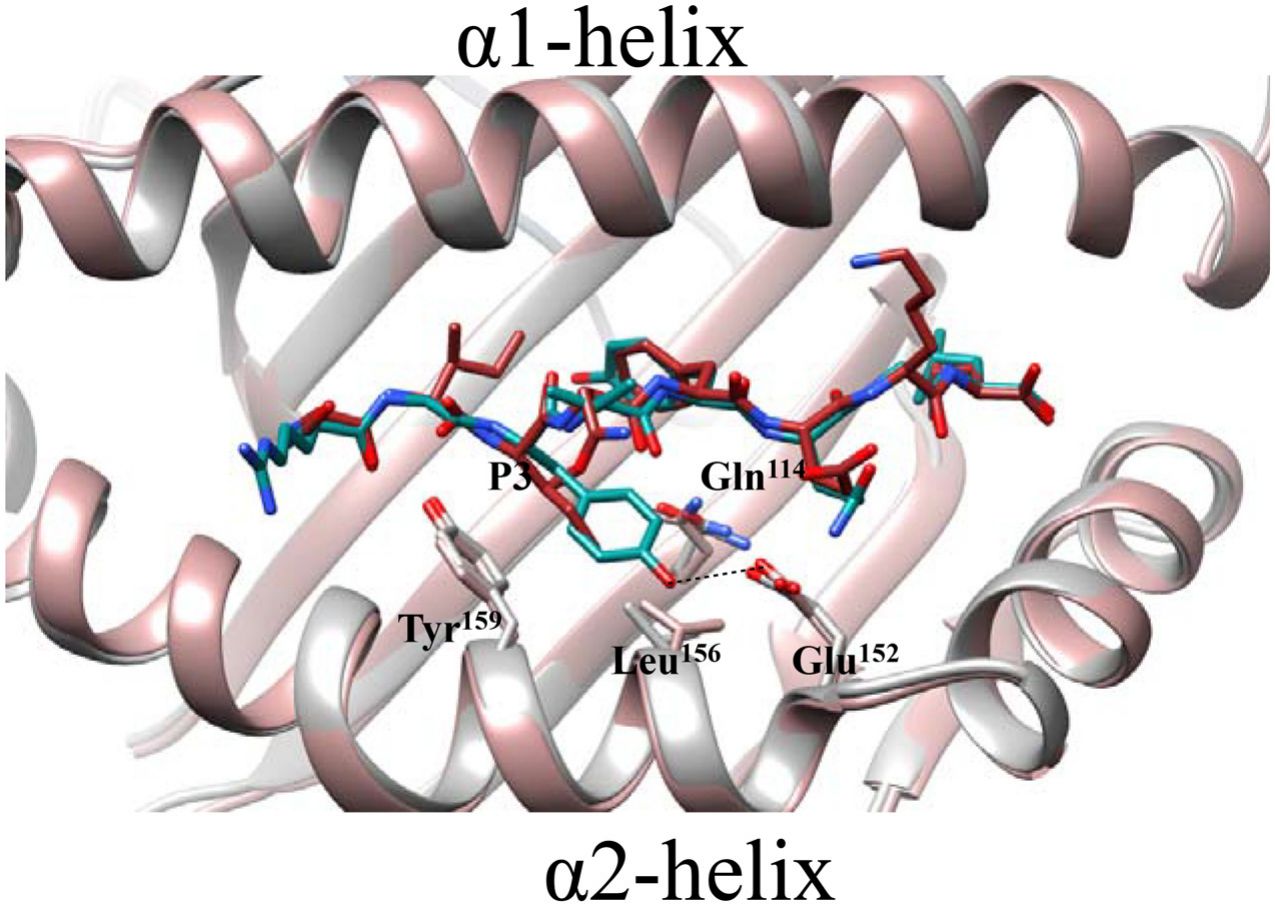
Structural analysis of peptide amino acid docking into the D-pocket of H-2K^b^. The auxiliary anchor residue at position 3 (P3) docks into the shallow D-pocket of H-2K^b^ formed by residues within the α2-helix. Comparison of P3 in H-2K^b^-SIINFEKL and H-2K^b^-RGYVYQGL, complexes that do or do not support Ly49C-H-2K^b^ interaction, respectively. The figure shows the H-2K^b^-SIINFEKL heavy chain in pink ribbon, the SIINFEKL peptide is in dark red; the H-2K^b^-RGYVYQGL heavy chain is in gray ribbon, the RGYVYQGL peptide is in teal. The figure was generated using CHIMERA UCSF software and PDB IDs IVAC for H-2K^b^-SIINFEKL and 1KPU for H-2K^b^-RGYVYQGL.

Analysis of the crystal structure of H-2K^b^ bound to SIINFEKL and RGYVYQGL can shed light into the possible molecular mechanism directing peptide specificity by the P3 residue. The P3 residue occupies the D-pocket which is formed by residues within the floor of the peptide binding groove as well as within the α2-helix: Gln^114^, Glu^152^, Leu^156^ and Tyr^159^ [34]. Interestingly, the D-pocket is located near the ‘hinge’ region joining the two segmented areas of the α2-helix, a structural component of the heavy chain that provides additional flexibility to accommodate different peptides [35]. Residue Tyr^159^ appears to have similar positioning in both structures, while Gln^114^ and Leu^156^ show slight displacement differences, however the greatest contrast is observed in Glu^152^ within the B-pocket (Fig 4). The bulky and polar amino acid at the auxiliary anchor residue P3, as with the RGYVYQGL peptide, occupies a larger area and gives rise to hydrogen bond formation with Glu^152^; while in the SIINFEKL peptide, the uncharged and relatively small size of Ile only results in van der Waals interactions with amino acids in the B-pocket. Interactions at the ‘hinge’ region of the α2-helix, including Glu^152^, can have an impact on solvent exposed residues in the loops that connect the antiparallel β-strands that form the platform of the groove floor, thereby possibly affecting Ly49C binding to H-2K^b^.

### P2 and P3 peptide residues combine to drive Ly49W/C recognition of H-2K^b^

**Fig 5:**
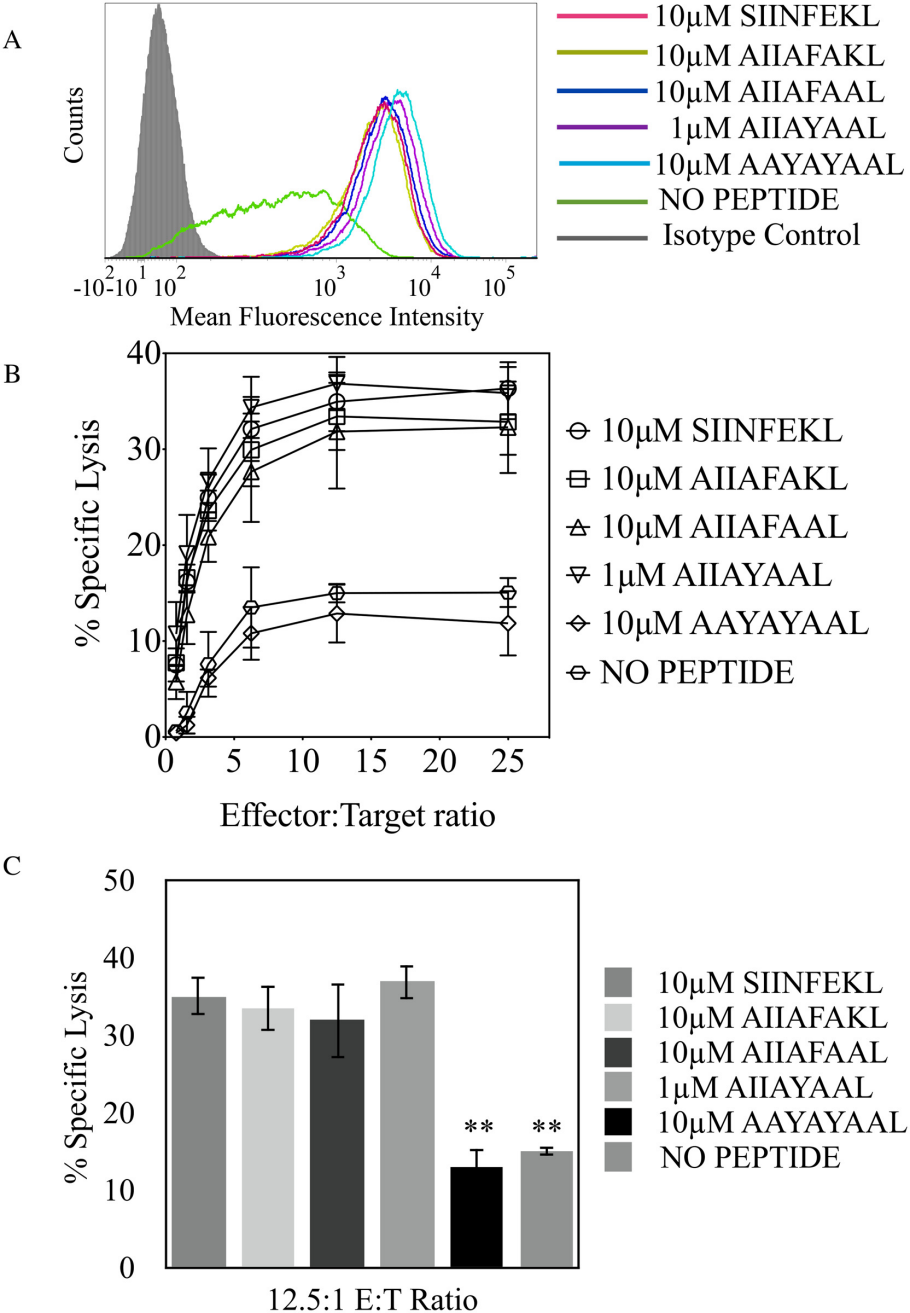
Peptide residues P2 and P3 determine Ly49C recognition of H-2K^b^. **(A)** Expression of H-2K^b^ incubated with the indicated peptides. **(B)** Percent cytotoxicity of RMA-S targets incubated with the indicated peptides incubated with RNK.49W/C effectors. **(C)** Statistically significant changes in Ly49W/C recognition of H-2K^b^, with respect to the positive control for RNK.49W/C recognition (SIINFEKL-loaded RMA/S cells), were conducted at the 12.5:1 E:T ratio from cytotoxicity assays (**p < 0.005). Results plotted are the mean of three independent experiments with error bars representing SD.

We further investigated the role of peptide residues in Ly49W/C recognition of H-2K^b^ by comparing variants of a synthetic peptide, AAYAYAAL. Like RGYVYQGL, AAYAYAAL is not supportive of H-2K^b^ interaction with Ly49W/C. We recapitulated the identity of SIINFEKL residues that we and others have found to contribute to Ly49C recognition into the AAYAYAAL peptide to test gain of recognition. Peptides used in this assay include AIIAFAKL, AIIAFAAL, and AIIAYAAL, that bind H-2K^b^ at similar levels, demonstrated by RMA/S stabilization assays that were performed in parallel to cytotoxicity assays (Fig 5A). Interestingly, the peptide AIIAFAKL is fully supportive of Ly49W/C recognition, as is AIIAFAAL and AIIAYAAL, suggesting a pivotal role for residues that dock into the adjacent B-pocket and D-pocket of H-2K^b^ which might be a site important to allow for H-2K^b^ and Ly49W/C interaction (Fig 5B and 5C). To pinpoint individual effects of P2 and P3 in Ly49W/C recognition of H-2K^b^, we tested P2 and P3 Ala substitutions in the AIIAYAAL peptide. However, peptides AAIAYAAL and AIAAYAAL did not stabilize H-2K^b^ on RMA-S cells for the duration of the cytotoxicity assays (S1 Fig). Nevertheless, our data indicate that specific amino acid identities at both P3 and P2 peptide residues are required for optimal H-2K^b^ recognition by Ly49W/C.

**Fig 6:**
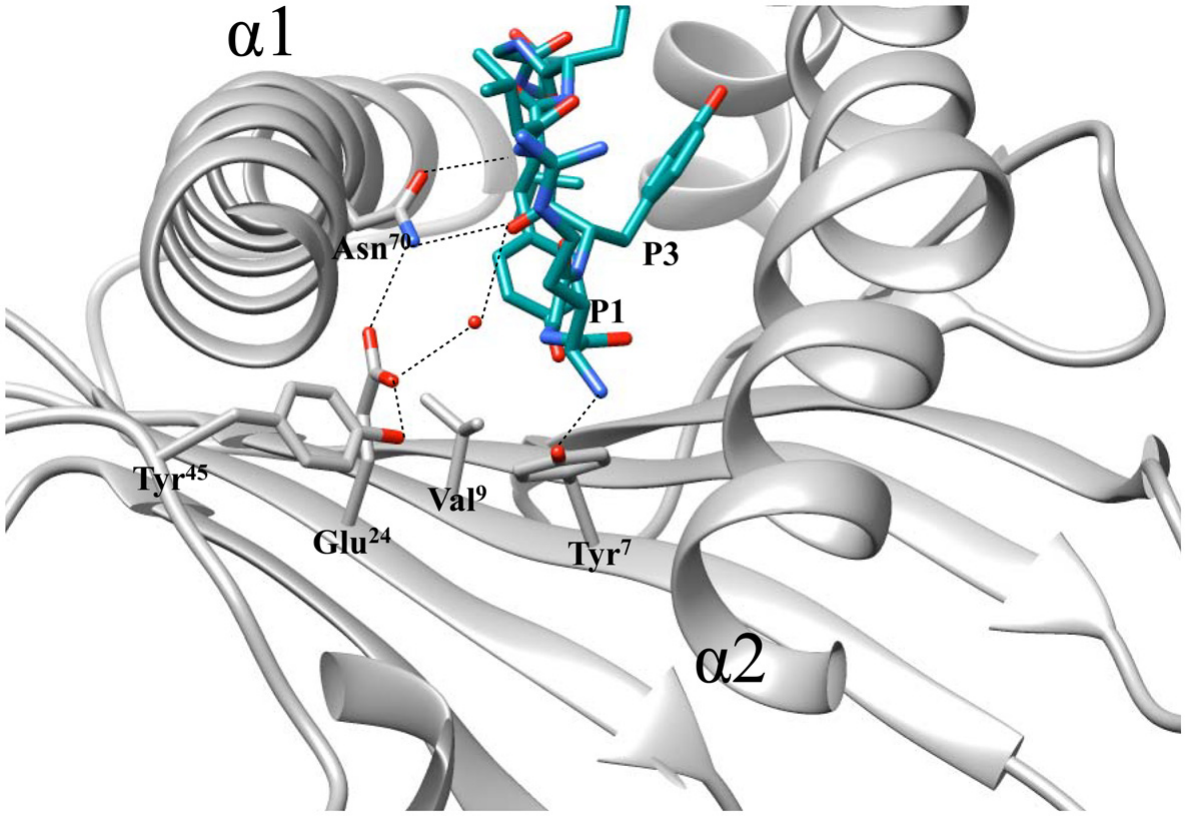
Structural analysis of peptide amino acid docking into the B-pocket of H-2K^b^. The H-2K^b^ bound peptide amino acid at position 2 (P2) docks into the B-pocket of H-2K^b^ in H-2K^b^-RGYVYQGL. Figure shows the H-2K^b^-RGYVYQGL heavy chain in gray ribbon, the RGYVYQGL peptide is in teal. The figure was generated using CHIMERA UCSF software and PDB ID 1KPU for H-2K^b^-RGYVYQGL.

From our experiments, in addition to P3, the P2 residue, that docks into the B-pocket formed by residues Tyr^7^, Val^9^, Glu^24^, Try^45^ and Asn^70^, is important in determining Ly49C and H-2K^b^ binding [34,36]. In the H-2K^b^-RGYVYQGL structure, Gly at P2 allows for a water molecule to fill the negatively charged B-pocket of H-2K^b^ and contribute to an extensive hydrogen bond network that stabilizes the core of the peptide-binding groove [35,36]. In the H-2K^b^-RGYVYQGL, this network involves the hydroxyl group of Tyr at P5 in the C-pocket and the α-carbon of P3 and a pseudobond with Glu^24^ within the B-pocket (Fig 6). On the other hand, with the H-2K^b^-SIINFEKL structure, Ile at P2 fully occupies the B-pocket, leaving no space for water molecules, possibly increasing the flexibility of the core of the peptide binding groove and decreasing the free energy requirements for Ly49C binding of H-2K^b^.

**Fig 7:**
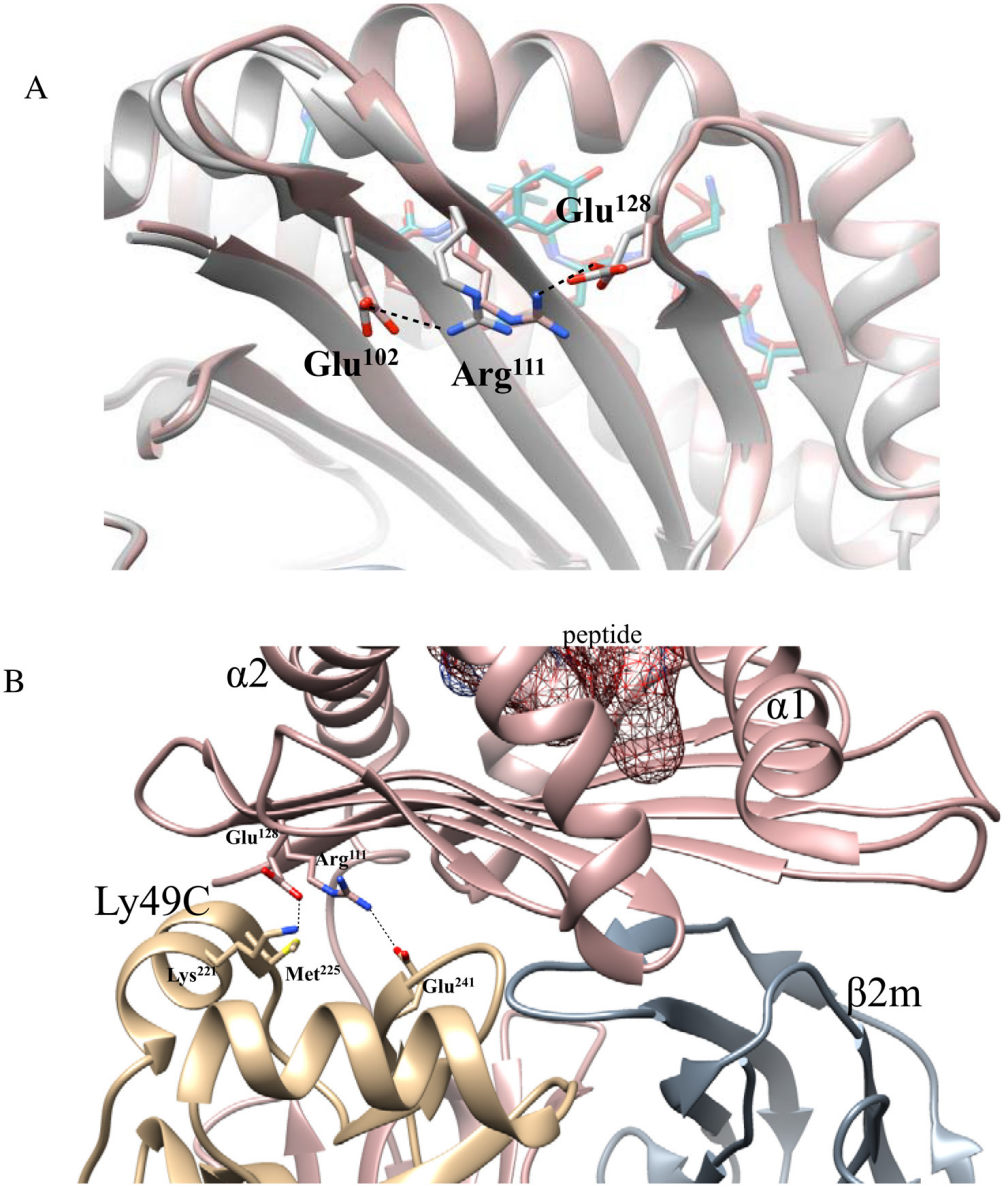
H-2K^b^ peptide dependent intramolecular interactions that potentially affect H-2K^b^ and Ly49C association. **(A)** Comparison between H-2K^b^-RGYVYQGL and H-2K^b^-SIINFEKL residues that participate in Ly49C interaction. Differences in intramolecular interactions within the H-2K^b^ that are affected by the peptide bound may be an important factor for the support of receptor and ligand association. Figure shows the H-2K^b^-SIINFEKL heavy chain in pink ribbon, the SIINFEKL peptide is in dark red; the H-2K^b^-RGYVYQGL heavy chain is in gray ribbon, the RGYVYQGL peptide is in teal. Hydrogen bonds are shown in black dashed lines. **(B)** Co-crystal structure of H-2K^b^-SIINFEKL and Ly49C. Figure shows the H-2K^b^-SIINFEKL heavy chain in pink ribbon, the β2m in blue ribbon and the SIINFEKL peptide in dark red. The CTLD of the Ly49C monomer is shown in gold ribbon. The figures were generated using CHIMERA UCSF software and PDB IDs IVAC for H-2K^b^-SIINFEKL, 1KPU for H-2K^b^-RGYVYQGL and 3C8K for H-2K^b^-SIINFEKL-Ly49C.

Further comparison of H-2K^b^ bound to SIINFEKL and RGYVYQGL shows different intramolecular interactions that can be of importance to Ly49C association. For example, in the H-2K^b^-SIINFEKL complex, Arg^111^ forms a hydrogen bond with Glu^128^, but in the H-2K^b^-RGYVYQGL complex Arg^111^ instead shares a hydrogen bond with Glu^102^ (Fig 7A). This difference in intramolecular interactions, may affect Ly49C binding, since Arg^111^ shares van der Waals contacts with Met^225^ in Ly49C, as well as salt bridges with Glu^241^ in Ly49C; and Glu^128^ forms salt bridges with Lys^221^ of Ly49C, as observed in the H-2K^b^-SIINFEKL-Ly49C co-crystal structure (Fig 7B). The aforementioned Met^225^ and Lys^221^, are part of the α3 helix in Ly49C, a structural motif that can be of importance in detection of the peptide bound to MHC-I, and contributes to H-2K^b^ recognition and binding affinity, while the peptide non-discriminating Ly49A receptor instead of forming an alpha helix at region L3, contains a disordered loop [14]. Although more receptors need to be examined for their peptide specificity, it would be interesting to define what determines an Ly49 to be peptide specific as opposed to one that is not. Studying the structural specificity of Ly49-MHC-I interactions can be of importance to understand the molecular basis of regulation controlling NK cell effector functions as well as NK cell development. In addition, and equally important, is the identification of natural peptides that are recognized as self by Ly49 molecules, when bound to MHC-I.

In addition to structural determinants affected by P2 and P3 residues, for Ly49C and H-2K^b^ association, amino acids at P2 and P3 can also influence complex stability that can translate into differences in peptide binding affinity for H-2K^b^. In our studies, all peptides tested stabilized H-2K^b^ on RMA/S cells for the duration of functional assays. Using a bioinformatics method, we predicted IC_50_ values for H-2K^b^ and natural peptides tested in this study, as an indirect indicator of peptide and H-2K^b^ affinity. The predicted IC_50_ values were obtained using the algorithm NetMHCpan which takes into account the identity of peptide residues and specific MHC allele information to produce quantitative predictions of the affinity between the peptide and the MHC of interest [21]. Most of the peptides that have strong predicted binding affinity for H-2K^b^ were in the group of peptides that did not support Ly49W/C recognition; while the majority of the predicted weaker H-2K^b^ peptide binders were also part of the set of peptides supporting Ly49W/C binding (Tables 4 and 5). It is possible that residues docking into the peptide binding groove near the N-terminal region of the peptide may provide more or less conformational flexibility of H-2K^b^ that, although correlating with overall affinity, also is associated with the ability or not to bind Ly49C. Moreover, the different peptide binding affinities could have an impact on NK cell recognition by Ly49 receptors.

**Table 4:** Predicted peptide binding affinity to H-2K^b^ for peptides supportive of Ly49W/C and H-2K^b^ interaction.

Peptide Sequence	Peptide origin	IC_50_ for association with H-2K^b^ (nM)
SI**I**N**F**EK**L**	Chicken Ovalbumin (256–264)	392.3601
KI**I**T**Y**RN**L**	PCI domain-containing protein 2 (318–325)	404.9105
KV**I**T**F**ID**L**	GTP binding protein I (246–253)	498.5564
YA**M**I**Y**RN**L**	E3 ubiquitin-protein ligase mdm2 (100–107)	126.4429

The predicted IC_50_ values for H-2K^b^and specific peptides that support Ly49W/C recognition of H-2K^b^ were obtained using the server NetMHCpan. Predictions were set up to have IC_50_ value of 10nM for strong affinity and IC_50_ value of 500nM for weak affinity. In the peptide sequence bold indicates primary anchor residue, bold and underlined indicates P3 auxiliary anchor residue.

**Table 5:** Predicted peptide binding affinity to H-2K^b^ for peptides that poorly, or do not, support Ly49W/C and H-2K^b^ interaction.

Peptide Sequence	Peptide origin	IC_50_ for association with H-2K^b^ (nM)
AN**Y**D**F**IC**V**	MMTV env gp70 (446–453)	254.4448
RG**Y**V**Y**QG**L**	Vesicular Stomatitis Virus NP (52–59)	96.0161
IS**F**K**F**DH**L**	F-actin capping protein alpha1 (93–100)	35.7493
IN**F**D**F**PK**L**	RNA helicase p54 (407–414)	62.7041
AA**Y**A**Y**AA**L**	Synthetic peptide	32.7871

The predicted IC_50_ values for H-2K^b^ and specific peptides were obtained using the server NetMHCpan. Predictions were set up to have IC_50_ value of 10nM for strong affinity and IC_50_ value of 500nM for weak affinity. In the peptide sequence bold indicates primary anchor residue, bold and underlined indicates P3 auxiliary anchor residue.

### Concluding remarks

Peptide selectivity of Ly49 recognition may afford NK cells an added level of surveillance beyond simply detecting the presence or absence of specific MHC-I allele products. This could be useful in the context of cell transformation or pathogenic infection, where the expression level of MHC-I might not change, but its peptide content may [11,12,37]. In this case, Ly49 may be able to discriminate MHC-I bound with different peptide repertoires, distinguishing “healthy self” from “non-self” and/or “non-healthy self” due to different bound peptides. Advances in mass spectrometric identification of MHC-I-bound peptides, combined with a greater understanding of peptide features that confer peptide specific recognition of MHC-I by Ly49 receptors as provided here, might facilitate prediction and manipulation of NK cell responses [37–39]. In addition, the importance of peptide selectivity *in vivo* remains to be established; for example, during NK cell education or target cell transformation, processes where Ly49-MHC-I interactions are fundamental to the fate and function of NK cells [4,40]. Additionally, inhibitory Ly49 and MHC-I interactions in cis, i.e. on the same NK cell, are an important part of NK cell regulation, where such engagement lowers the threshold for NK cell activation, and can also play a role during NK cell education [41,42]. However, whether Ly49 interaction with MHC-I in cis is peptide selective, or if peptide selectivity is restricted to trans Ly49 interaction, has not been demonstrated. Furthermore, it remains to be determined how many more receptors in the Ly49 family are peptide selective in ligand recognition, including activating Ly49 that can recognize MHC-I [41–43]. Finally, identifying anchor residues as prime drivers of Ly49 peptide selectivity, and the endogenous peptides within which they reside, can be of importance towards defining self for NK cells.

## Supporting Information

S1 Fig H-2K^b^ stabilization using AAYAYAAL peptide variants to recapitulate SIINFEKL anchor residues. RMA/S stabilization assay using 10μM of the indicated peptide. Cell surface expression of H-2K^b^ was detected using the APC conjugated AF6-88.5.5.3 antibody. Assays were conducted in triplicate with error bars indicating SD.
(EPS)
